# Insights into the molecular mechanism underlying CD4-dependency and neutralization sensitivity of HIV-1: a comparative molecular dynamics study on gp120s from isolates with different phenotypes†

**DOI:** 10.1039/c8ra00425k

**Published:** 2018-04-17

**Authors:** Yi Li, Lei Deng, Shi-Meng Ai, Peng Sang, Jing Yang, Yuan-Lin Xia, Zhi-Bi Zhang, Yun-Xin Fu, Shu-Qun Liu

**Affiliations:** State Key Laboratory for Conservation and Utilization of Bio-Resources in Yunnan, Yunnan University Kunming P. R. China Yunxin.Fu@uth.tmc.edu shuqunliu@ynu.edu.cn; Department of Applied Mathematics, Yunnan Agricultural University Kunming P. R. China; College of Agriculture and Biological Science, Dali University Dali P. R. China; Human Genetics Center and Division of Biostatistics, School of Public Health, The University of Texas Health Science Center Houston USA

## Abstract

The envelope (Env) of HIV-1 plays critical roles in viral infection and immune evasion. Although structures of prefusion Env have been determined and phenotypes relevant to the CD4 dependency and the neutralization sensitivity for various HIV-1 isolates have been identified, the detailed structural dynamics and energetics underlying these two phenotypes have remained elusive. In this study, two unliganded structural models of gp120, one from the CD4-dependent, neutralization-resistant isolate H061.14 and the other from the CD4-independent, neutralization-sensitive R2 strain, were constructed, and subsequently were subjected to multiple-replica molecular dynamics (MD) simulations followed by free energy landscape (FEL) construction. Comparative analyses of MD trajectories reveal that during simulations R2-gp120 demonstrated larger structural fluctuations/deviations and higher global conformational flexibility than H061.14-gp120. Close comparison of local conformational flexibility shows that some of the structural regions involving direct interactions with gp41 and adjacent gp120 subunits in the context of the closed trimeric Env exhibit significantly higher flexibility in R2-gp120 than in H061.14-gp120, thus likely increasing the probability for R2-Env to open the trimer crown and prime gp41 fusogenic properties without induction by CD4. Collective motions derived from principal component analysis (PCA) reveal that R2-gp120 is prone to spontaneous transition to the neutralization-sensitive CD4-bound state while H061.14-gp120 tends to maintain the neutralization-resistant unliganded state. Finally, comparison between FELs reveals that R2-gp120 has larger conformational entropy, richer conformational diversity, and lower thermostability than H061.14-gp120, thus explaining why R2-gp120 is more structurally unstable and conformationally flexible, and has a higher propensity to transition to the CD4-bound state than H061.14-gp120. The present results reveal that the differences in dynamics and energetics between R2-gp120 and H061.14-gp120 impart Env trimers with distinct capacities to sample different states (*i.e.*, R2-Env samples more readily the open state while H061.14-Env is more inclined to maintain the closed state), thus shedding light on the molecular mechanism underlying the HIV-1 phenotype associated with CD4 dependency/neutralization sensitivity.

## Abstract 2

The envelope (Env) of HIV-1 plays critical roles in viral infection and immune evasion.

## Graphical abstract

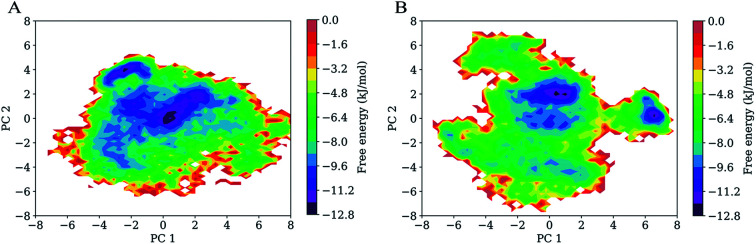

## Introduction

As the fusion machinery of human immunodeficiency virus type 1 (HIV-1), the trimeric envelope (Env) composed of three gp120-gp41 glycoprotein heterodimers, plays critical roles in viral entry and immune evasion.^1–4^ Structural studies^5–9^ and biophysical assays^10–12^ have revealed that Env evades antibody-mediated neutralization by favouring a closed, ground-state conformation, in which the variable regions 1 and 2 (V1/V2) of gp120 shield the variable loop 3 (V3), the inter-protomer interactions occurring among V1/V2 and V3 of gp120 subunits lock the trimer apex/crown, and the bridging sheet participating in the formation of coreceptor-binding site (*i.e.*, for CCR5 or CXCR4) is absent due to the difference in orientations of β2 and β3 relative to β20-β21 hairpin between the unliganded and CD4-bound forms of gp120.^13,14^ Interactions with the initial receptor, CD4, induce substantial changes in gp120 conformation,^15^ leading to the opening of the trimer crown and the formation and exposure of the coreceptor-binding site (*i.e.*, bridging sheet and V3 loop),^16,17^ to which the binding of coreceptor triggers additional conformational rearrangements of gp41 to form a stable six-helix bundle that facilitates the fusion between viral and cellular membrane.^18^ Because Env in the unliganded, closed state and in the CD4-bound, open state masks and exposes conserved epitopes for certain neutralizing antibodies (*e.g.*, CD4-induced (CD4i) antibody 17b and CD4-binding-site (CD4bs) antibody b12), respectively, these two forms of Env display distinct susceptibilities to neutralization by these antibodies, *i.e.*, the open Env is more readily to be neutralized than the closed one.^12,19^

Although HIV-1 infection and Env immunization elicit an abundant production of Env-directed antibodies, many of them cannot completely suppress virus replication in infected hosts due to the effective strategies of HIV-1's immune evasion, including the rapid genetic variation,^20,21^ the glycan shield,^22–24^ and the conformational masking,^10^ all of which enable HIV-1 to escape neutralizing antibodies. In particular, the latter two originate from steric hindrance effects that act through concealing the functional centres or hiding vulnerable shape/sites of Env, especially those on gp120, from attack by antibodies.^25^

Through examining plasma from patients with acute HIV-1 infection, Wei *et al.*^24^ found that the viral inhibitory activity of neutralizing antibodies resulted in complete replacement of neutralization-sensitive virus with successive populations of neutralization-resistant virus. Furthermore, although most primary isolate strains have traditionally been considered resistant to neutralizing antibodies, there is still a wide spectrum of neutralization sensitivity among isolated circulating Envs.^26^ Under the immune selection pressure, the primary, clinical HIV-1 isolates, especially those (“tier 2” or “tier 3” viruses) displaying a more neutralization-resistant phenotype, infect the host cell strictly depending on interactions with CD4.^27–29^ However, some primary HIV-1 isolates, which can infect macrophages and brain microglia cells that express only low levels of CD4, exhibit reduced CD4 dependence for virus entry and enhanced sensitivity to neutralization by antibodies.^30,31^ Binding of CD4 is not an integrant step in infection by the laboratory-adapted, CD4-independent isolates, which were passaged on CD4-negative, coreceptor-positive cells in culture medium lacking antibody selective pressure and hence evolved to be more sensitive to antibody-mediated neutralization than the primary clinical isolates.^32–34^ To this end, the HIV-1 isolates that infect cells in the CD4-independent manner are characterized by high sensitivity to neutralizing antibodies, while the isolates with the high neutralization resistance are all CD4 dependent, indicating that the phenotypes of CD4 dependency and neutralization sensitivity are reversely correlated. For clarity, in this study we define the CD4-independent infectivity and the high neutralization sensitivity as the same equivalent phenotype, and CD4-dependent infectivity and high neutralization resistance as another same phenotype. Of note is that the CD4-independent, neutralization-sensitive isolates have evolved mutations that could stabilize some specific prefusion states (*e.g.*, open conformations) of Env that allow for the high-affinity interaction with coreceptor when CD4 is either present at low levels or entirely absent.^12,34^

The conformational rearrangements within gp120 not only provide the structural basis for receptor-mediated HIV-1 entry, but also affect viral sensitivity to antibody-mediated neutralization. Both experimental^16,35^ and theoretical studies^36,37^ have demonstrated that the binding of CD4 to gp120 results in the disruption of contacts between V1/V2 and V3 at the trimer apex accompanied by large repositioning of these regions, rearrangements of the bridging-sheet elements, and formation and exposure of the coreceptor-binding site, ultimately leading to an open CD4-bound state of Env trimer. The V1/V2 region has been shown to play a critical role in regulating the neutralization phenotypes of primary HIV-1 isolates,^38^ probably *via* destabilizing the inter-protomer associations at the apex of the trimeric Env.^12,39^ Comparative molecular dynamics simulations of the unliganded gp120 monomer and gp120-18A (a compound identified as a broad-spectrum anti-HIV inhibitor) complex revealed that 18A inhibited virus entry through maintaining the unliganded state of gp120 by impeding rearrangements of the V1/V2 region.^40^ Single-molecule fluorescence resonance energy transfer (smFRET) analysis has revealed that (i) the ligand-free Env trimers on the surface of HIV-1 virions coexist in three distinct prefusion conformations (*i.e.*, the most populated, unliganded closed ground state; the moderately populated, open activation state that resembles the CD4/17b-stabilized conformation, and the least populated, relatively stable intermediate state during the transition from the closed to the open state) whose relative populations can be remodeled by CD4 and 17b binding, and (ii) the ligand-free Env trimers from the neutralization-sensitive isolate NL4-3 frequently transition out of the closed state and hence exhibit a higher occupancy of the open state than those from the neutralization-resistant isolate JR-FL.^11^ Therefore, the ligand-free Env is able to spontaneously transition among different states in the absence of receptor induction and the difference in the conformational transition capability (or relative occupancies of the states) between Envs is responsible for differential phenotypes of neutralization sensitivity and resistance among HIV-1 isolates.

Currently, more and more atomic-level structures of the HIV-1 Env in various conformational states have been achieved,^5–8,41^ and these structures provide a more complete and comprehensive picture of the molecular mechanisms underlying HIV-1 entry into cells and escape from antibody neutralization. In addition, different subgroups of HIV-1 isolates that represent distinct categories of neutralization sensitivity have also been identified.^26^ However, the detailed questions about what features in gp120 structure dictate the capability of gp120/Env to undergo conformational changes, and how the dynamic behaviour of gp120 determines the distinct phenotypes of neutralization sensitivity/CD4 dependency of HIV-1, have remained unanswered.

In order to probe the relationship between the structural dynamics of gp120 and the viral phenotype of neutralization sensitivity/CD4 dependency, in this paper, two near-full-length gp120 structural models in the unliganded state, one from the CD4-dependent, neutralization-resistant HIV-1 isolate H061.14 (hereafter referred to as H061.14-gp120) and the other from the CD4-independent, neutralization-sensitive R2 strain (R2-gp120), were built using homology modelling method. Subsequently, these two models were subjected to molecular dynamics (MD) simulations followed by principal component analysis (PCA) and free energy landscape (FEL) reconstruction to investigate the differences in structural stability, conformational flexibility, molecular motions, and FELs between them. Previous smFRET analyses^11^ showed that although the ligand-free Env/gp120 from a neutralization-sensitive isolate NL4-3 has a larger population of CD4-bound, open conformation than that from a neutralization-resistant HIV-1 JR-FL, the unliganded, closed state is still the dominant conformation for both ligand-free Envs/gp120s; furthermore, although the CD4-bound, open conformation becomes dominant upon binding of CD4/17b, the unliganded, closed conformation still exists for both Envs/gp120s, with smaller population observed for NL4-3-Env/gp120. In addition, the reliable template (or experimental structure) for modeling the full-length structural model of gp120 in the CD4-bound form is still not available. Therefore in this paper we focus on comparing the dynamical properties and conformational transition capability between phenotypically different gp120s only in their unliganded form. The results reveal that the unliganded model of R2-gp120 is more structurally unstable, conformationally flexible, and prone to spontaneous transition to the CD4-bound state than the unliganded H061.14-gp120, thus providing a reasonable explanation for why isolates H061.14 and R2 show the phenotypic difference in neutralization sensitivity/CD4 dependency.

## Materials and methods

### Sequence preparation

In order to minimize the background noise and improve the validity of comparisons, gp120 sequences from two primary HIV-1 isolates belonging to the same clade (clade B) but with significant difference in the phenotype of neutralization sensitivity/CD4 dependency, *i.e.*, H061.14 ^26^ and R2,^42^ were used as target sequences for homology modelling. H061.14, a sexually transmitted chronic isolate, is more neutralization resistant and as such was categorized as the “tier 3” virus.^26^ R2 strain, isolated from a donor with long-term non-progressive HIV-1 infection, is more neutralization sensitive, cross-reactively neutralized by various HIV-immune human sera, and capable of utilizing the coreceptor CCR5 in the CD4-independent manner for cell entry.^43^ The gp160 sequences of H061.14 and R2 isolates were obtained from UniProtKB database (http://www.uniprot.org), with accession numbers being A4ZPW8 and Q9WPZ4, respectively. For both gp160 sequences, the segments corresponding to the signal peptide and gp41, as well as a part of gp120 N-terminal residues, were removed. The finally obtained sequences of H061.14-gp120 and R2-gp120 comprise 467 (residues 31–497) and 479 (residues 31–509) amino acid residues, respectively, and have a sequence identity of 71%.

### Homology modelling

The homology-modelling procedure implemented in MODELLER version 9.17 ^44^ was used to build the structural models of H061.14- and R2-gp120 in the unliganded state. The gp120 atomic coordinate extracted from the crystal structure of HIV-1 X1193.c1 SOSIP.664 prefusion Env trimer, which was obtained from Protein Data Bank (PDB) (http://www.rcsb.org) with PDB ID 5FYJ (chain G) at 3.4 Å resolution,^6^ was used as the template. It should be noted that the full-length, atomic-resolution structure of monomeric gp120 in the ligand-free state is still currently unavailable. However, small-angle X-ray scattering data have revealed that the full-length structure of gp120 free in solution closely resembles that of gp120 subunit in the context of the oligomeric viral spike/trimer.^35^ Therefore, it is reasonable to use gp120 subunit structure extracted from a trimeric Env as the template for constructing the monomeric gp120 structural model.

**Fig. 1:**
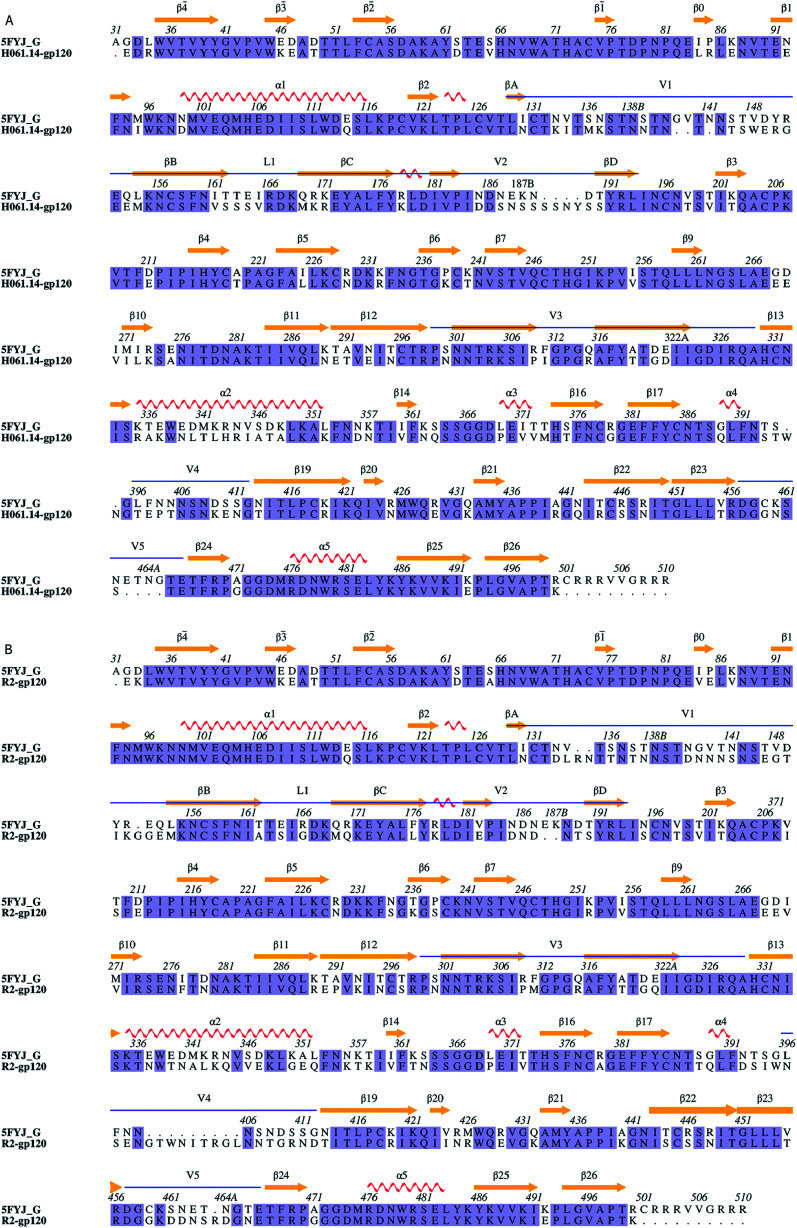
Sequence alignments between the target and the template. (A) and (B) Are sequence alignments used for building structural models of H061.14-gp120 and R2-gp120, respectively. 5FYJ_G represents the template sequence from the crystal structure with PDB ID 5FYJ (chain G). Residues are numbered according to the template sequence. Conserved residues are shaded in light blue. Regular secondary structural elements of the template are numbered according to HXBc2 crystal structures (PDB IDs: 3JWD and 1G9M),^15,48^ with orange arrows and red spirals representing β-strands and α-helices (or 3/10 helices), respectively. The variable regions, *i.e.*, V1/V2, V3, V4, and V5 are indicated above the alignment by blue line segments. The four β-strands in the V1/V2 region, designated A to D,^49^ are labelled βA to βD, respectively. V1 and V2 loops are located between βA and βB and between βC and βD, respectively, and the loop connecting βB and βC is labelled L1.

Sequence alignments of the two targets relative to the template were shown in Fig. 1. The high sequence identity of H061.14-gp120 and R2-gp120 with respect to the template (71% and 70%, respectively) guarantees the reliability of the constructed structural models. For each of these two gp120s, 20 structural models were generated and only the one with the lowest molecular probability density function score was selected. Subsequently, the two final gp120 models were validated using programs PROCHECK,^45^ PROVE^46^ and VERIFY3D^47^ available in SAVES server (https://services.mbi.ucla.edu/SAVES/). The result of PROCHECK revealed that for both models, ∼91% of residues fell within the most favoured regions while only 0.5% of residues were in the disallowed regions of the Ramachandran plots (ESI Fig. S1†), indicating a good stereochemical quality of these two models. The structural qualities in terms of PROVE and VERIFY3D were comparable to that of the template used (ESI Table S1†), indicating that these two gp120 models were suitable for further structural analysis and MD simulations.

### MD simulations

Before MD simulations, the two structural models were individually solvated using TIP3P water model^50^ in a dodecahedron box with a solute-wall minimum distance of 0.8 nm. To obtain the electroneutral system with a salt concentration of 150 mM, 130 Cl^−^ and 127 Na^+^ and 132 Cl^−^ and 130 Na^+^ were introduced to protein-solvent systems of H061.14-gp120 and R2-gp120, respectively, adding up to a total number of atoms of 131, 427 and 134, 526, respectively.

All simulations were performed by employing GROMACS 5.1.4 ^51^ package with the AMBER99SB-ILDN^52^ force field. Initially, each system was subjected to energy minimization with steepest descent algorithm until no significant energy change could be detected. Then, the systems were simulated by four successive 200 ps position-restrained MD runs with decreasing harmonic positional restraint force constants on the protein heavy atoms (Kposres = 1000, 100, 10 and 0 kJ mol^−1^ nm^−2^). Finally, production MD runs were conducted for each system with the following protocols used: LINCS algorithm^53^ was used to constrain bond lengths so that an integration time step of 2fs can be adopted; long-range electrostatic interactions were treated using the particle-mesh Ewald (PME) method^54^ with a fourth-order interpolation, Fourier grid spacing of 0.135 nm, and coulomb radius of 1.0 nm; A twin-range cut-off was used for calculation of van der Waals (VDW) interactions, with the short- and long-range cut-off distances set to 1.0 and 1.4 nm, respectively; the non-bonded pair list was updated every 10 time steps; structural frames were saved every 2 ps; protein and non-protein (solvent and ions) components were independently coupled to a 300 K heat bath with a coupling constant *τ*_t_ of 0.1 ps; and the pressure was maintained by weakly coupling the system to an external pressure bath at 1 atm with a coupling constant *τ*_p_ of 0.5 ps.^55^ In order to sample the conformational space more efficiently, for each system, eight 30 ns production MD simulations, each starting with different initial atomic velocities assigned from Maxwell distribution at 300 K, were performed.

### Analysis methods

The tools ‘gmx rmsd’ and ‘gmx rmsf’ within GROMACS were used to calculate the backbone root-mean-square deviation (RMSD) and the C_α_ root-mean-square fluctuations (RMSF), respectively. The collective motions of gp120s were filtered by principal component analysis (PCA), which was performed through diagonalization of the covariance matrix built from C_α_ atomic fluctuations in a MD trajectory. The obtained eigenvectors/principal components (PC) and corresponding eigenvalues are representatives of the collective motion modes of a protein structure and the amplitudes of atomic fluctuations along eigenvectors, respectively. Collective modes of protein motions along the eigenvectors 1 and 2 were shown as the porcupine plots, which were obtained using a modevectors py script implemented in PyMol (http://www.pymol.org) with the two extremes extracted from the eigenvector projections as input. PCs 1 and 2 were chosen as reaction coordinates to construct FELs by using probability density function *F*(*s*) = −*k*_B_*T* ln(*N*_*i*_/*N*_max_), where *k*_B_ is Boltzmann's constant, *T* is the temperature of simulation systems, *N*_*i*_ is the population of bin *i*, and *N*_max_ is the population of the most populated bin.

For purpose of clarity, residue numbering of both gp120s was according to that of the template sequence; regular secondary structural elements were numbered according to the convention in reference to the HXBc2 crystal structures (PDB IDs: 3JWD and 1G9M).^15,48^ The structurally equivalent residue position was determined from the structure-based multiple sequence alignment among the template, H061.14- and R2-gp120 (ESI Fig. S2,† which was obtained using the Dali server (http://ekhidna2.biocenter.helsinki.fi/dali/).^56^

## Results and discussion

### Structural models

**Fig. 2:**
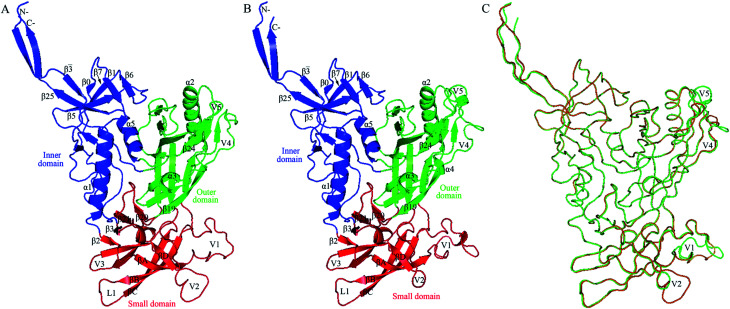
Structural models of the two near full-length gp120s in the unliganded state. (A) Ribbon representation of the model of H061.14-gp120. (B) Ribbon representation of the model of R2-gp120. (C) Backbone superposition of the models of H061.14-gp120 (orange) and R2-gp120 (green). In (A) and (B), the inner domain, outer domain, and small domain (composed of V1/V2, V3, and bridging-sheet elements β2-β3 and β20-β21) are coloured blue, green, and red, respectively; secondary structural elements in the inner and outer domains are numbered in reference to crystal structures of HXBc2 gp120 core (PDB IDs: 3JWD and 1G9M);^15,48^ the “Greek key”-like V1/V2 in the small domain is composed of four antiparallel β-strands (labelled βA to βD) and three connecting loops: V1 (between βA and βB), L1 (between βB and βC), and V2 (between βC and βD); the two bridging-sheet elements, β2-β3 and β20-β21, arrange in the order β2-β3-β21-β20 where β3 and β21 form a parallel β-sheet, in contrast to the order β3-β2-β21-β20 where β2 and β21 form an anti-parallel β-sheet observed in the CD4-bound state of gp120.^15,17^ The seven-stranded β-sandwich is composed of β3̄, β0, β1, β5, β6, β7, and β25; the layers 1 to 3, which emanate from the β-sandwich, are defined as the excursions between β3̄ and β0, between β1 and β5, and between β7 and β25, respectively.^15^

Fig. 2 shows the structural models of H061.14- and R2-gp120. Like the template, both models are composed of an inner domain, an outer domain, and a relatively small domain that lies beneath the juxtaposed inner and outer domains (Fig. 2A and B). The inner domain mainly consists of the N-, C-termini, a near-terminal seven-stranded β-sandwich (composed of β3̄, β0, β1, β5, β6, β7, and β25), and two α-helices (α1 and α5); the outer domain is primarily composed of two end-to-end stacked β-barrels, one α-helix (α2), and two loop excursions (V4 and V5); and the small domain comprises the V1/V2 region extended from the inner domain, the loop V3 extended from the outer domain, and the two elements (*i.e.*, V1/V2 stem composed of β2 and β3, and β20-β21 hairpin) of the bridging sheet. Of note is that in the unliganded state, the “Greek key”-folded V1/V2 ^49^ covers the V3 loop, which in turn packs against the two bridging-sheet elements that arrange in the order of β2-β3-β21-β20; therefore, in this state the coreceptor-binding site composed of the extended V3 and the β3-β2-β21-β20-arranged bridging sheet,^15,17^ is buried and has yet to be formed. Since the same template structure was used, the backbone RMSD between the two structural models is as small as 0.21 Å. The superimposition of these two models shows that much of the structure, especially the regular secondary structural elements, is well matched, with the exception of the N-, C-termini and some loops such as V1, V2, V4, and V5 displaying distinct conformations (Fig. 2C). This is not surprising, as insertions or deletions exist between amino acid sequences of these loops (ESI Fig. S2†) and, moreover, the surface-exposed loops usually display high flexibility and alternative conformations due to their direct interactions with the solvent.^57^

Through binding antigenicity analyses of a near-native Env trimer mimic, BG505 SOSIP.664, Kwon *et al.*^8^ have shown that (i) V3-directed (such as 447-52D and 3074) and bridging-sheet-directed antibodies (such as 17b) bind poorly to this Env mimic in the absence of CD4; (ii) the presence of CD4 significantly increases binding affinity of these antibodies; (iii) these antibodies cannot bind the 201C–433C double-cysteine Env mutant (DS-SOSIP.664) even in the presence of CD4. These results indicate that (i) the V3 and bridging-sheet epitopes are not accessible or not formed when gp120 is in the unliganded state, as shown in Fig. 2; (ii) CD4 binding exposes relevant epitopes *via* inducing conformational changes in gp120/Env; (iii) the disulfide bond (201C–433C) between β3 and β21 prevents rearrangement of the bridging-sheet elements from forming the mature bridging sheet and hence locks Env trimer in the closed, unliganded state. Moreover, the reason for BG505 SOSIP.664 to resist neutralization by 17b and relevant antibodies has been attributed to its dominant occupation of the closed unliganded conformation due to the weak conformational transition capability.^12^ Therefore, it can be anticipated that the different conformational transition capabilities of gp120/Env among different isolates are a major determinant of the neutralization sensitivity. In the following sections the dynamical properties of R2- and H061.14-120 will be analysed to probe the difference in the conformational transition capability between them.

### Structural stability during simulations

**Fig. 3:**
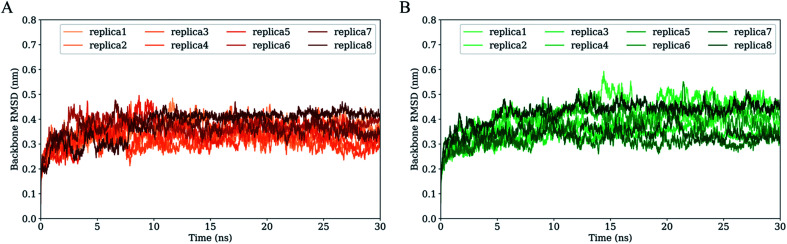
Time evolution of the backbone root-mean-square deviation (RMSD) values of the two gp120 structural models with respect to their respective starting structures during multiple-replica MD simulations. (A) RMSD curves of H061.14-gp120. (B) RMSD curves of R2-gp120. RMSD curves calculated from MD replicas 1 to 8 are shown in different colours.

Fig. 3 shows the time evolution of RMSD values of H061.14- and R2-gp120 relative to respective starting structures. Owning to large systems, each replica of both systems requires ∼5 ns to reach relatively stable RMSD values. After equilibrium, most replicas of H061.14-gp120 fluctuate around ∼0.35 nm and demonstrate small fluctuation amplitudes (Fig. 3A), whereas replicas of R2-gp120 exhibit a wider RMSD range (from ∼0.3 and ∼0.5 nm) and larger amplitudes of the RMSD fluctuation. Therefore, the neutralization-sensitive, CD4-independent R2-gp120 experienced larger structural deviations/conformational changes than the neutralization-resistant, CD4-dependent H061.14-gp120 during MD simulations, indicating that R2-gp120 has a lower structural stability. It is widely accepted that the lower the structural stability of a protein is, the higher its capability to alter its conformation, and *vice versa*. Therefore, the observed lower structural stability of R2-gp120 likely implies its higher capability of transitioning out of the unliganded state when compared to H061.14-gp120. It has been shown that the BG505 DS-SOSIP.664 mutant, which resists CD-induced conformational changes and cannot be neutralized by 17b, is more structurally/thermally stable than the parent SOSIP.664 trimer,^8^ supporting the viewpoint that structural stability is inversely correlated with conformational variability.

For each simulation system, the equilibrium portions (5–30 ns) of each replica were concatenated together to obtain a single 200 ns joined trajectory, which is the representative of different sampling directions around the starting structure. To ensure that the calculated parameters reflect the intrinsic properties of gp120s, all subsequent analyses were performed based on the two joined equilibrium trajectories.

### Conformational flexibility

**Fig. 4:**
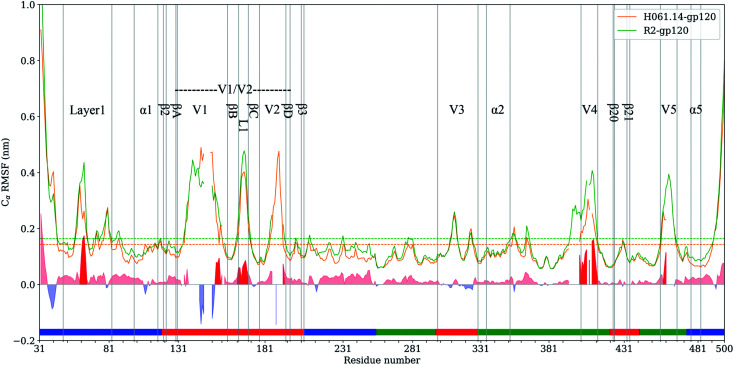
C_α_ atom root-mean-square-fluctuation (RMSF) profiles of H061.14-gp120 and R2-gp120 and their RMSF-difference as a function of residue number. C_α_ RMSF values of H061.14-gp120 (orange line) and R2-gp120 (green line) were calculated from respective joined equilibrium MD trajectories. Residue numbering is according to the template sequence (PDB ID: 5FYJ, chain G). Residues belonging to the inner domain (residues 31–119, 204–255, and 474–510), outer domain (residues 256–298, 330–421,443–473), and small domain (residues 120–203, 299–329, 422–442) are indicated above the horizontal axis by line segments coloured in blue, green, and red, respectively. The average RMSF values are shown as dotted lines. RMSF-difference was obtained by subtracting H061.14-gp120's RMSF values from R2-gp120's values at structurally equivalent residue positions, which were determined from the structure-based multiple sequence alignment shown in ESI Fig. S2.† The regions with RMSF-difference greater and less than 0 are shaded in light red and light blue, respectively, and the regions with RMSF-difference greater than 0.06 nm are highlighted in red.

RMSF values of C_α_ atoms, which are usually used as the flexibility index of protein structure, were computed based on the single joined equilibrium trajectories of these two gp120s. The RMSF values averaged over all C_α_ atoms of H061.14- and R2-gp120 are 0.14 and 0.16 nm (Fig. 4, dotted line), respectively, indicating that R2-gp120 has a higher global conformational flexibility than H061.14-gp120. As shown in Fig. 4, the two gp120s have similar RMSF features to each other, with the regular secondary structure regions, especially those in the outer domain, exhibiting the low RMSF values (lower than average values), while the N-, C-termini and surface-exposed loops (*e.g.*, layer 1, loops V1, L1, V2, V3 tip, V4, and V5) showing the high RMSF values (higher than average values). These results are consistent with the hydrogen/deuterium exchange (HDX) profile of the full-length monomeric gp120 from SF162 isolate,^35^ indicating that on the one hand MD simulations can provide reliable information about protein dynamics, on the other hand the full-length ligand-free gp120s from different isolates have similar distributions of the local conformational flexibility and rigidity. Nevertheless, close inspection of Fig. 4 reveals that most of structural regions are characterized by higher RMSF values in R2-gp120 than in H061.14-gp120. The quantitative measurement of the flexibility difference was conducted by subtracting RMSF values of H061.14-gp120 from those of R2-gp120 at structurally equivalent residue positions (shaded region in Fig. 4). It is clear that most of the structural regions, including those from either the inner and outer domains or the small domain, have higher conformational flexibility in R2-gp120 than in H061.14-gp120 (RMSF-difference > 0; shaded (light) red in Fig. 4); whereas only very few regions (*e.g.*, parts of the N-terminus and V1 loop) have apparently higher flexibility in H061.14-gp120 (RMSF-difference < 0 nm; shaded blue in Fig. 4). These explain why R2-gp120 has a higher global conformational flexibility (*i.e.*, higher average RMSF value) than H061.14-gp120.

The structural regions exhibiting significantly higher flexibility in R2-gp120, which are defined as those with RMSF-difference greater than 0.06 nm (shaded red in Fig. 4), contain residues 31–35 (a portion of the N-terminus), 58–64 (a portion of the layer 1), residues 143–147 and (a portion of V1 loop), 162–168 (the L1 loop connecting βB and βC in V1/V2), 393–412 (V4 loop), 458–460 (a portion of V5 loop).

The layer 1 in the inner domain of gp120 not only directly interacts with the N-terminal part of heptad repeat 1 (HR1) of gp41 ^6,13–15^ in the context of trimeric Env, but also packs against α1 in the layer 2 of gp120.^15^ It has been proposed that CD4-induced changes in gp120 conformation can be transmitted through α1 and layer 1 to HR1, thus facilitating the formation of the gp41 prehairpin intermediate for fusion proceeding.^15,16^ We consider here that, when compared to H061.14-gp120, the higher flexibility of layer 1 in R2-gp120 may be more conductive to triggering the gp41-fusion machinery even in the absence of CD4 because of the intimate contact between gp120 layer1 and gp41 HR1 in the trimeric context. In the V1/V2 region, although relative large RMSF-differences can be observed in residues 138–138C, 140–142, and 143–147 (parts of V1 loop located between βA and βB) and 188–189 (a portion of V2 loop located between βC and βD) between R2-gp120 and H061.14-gp120, these differences appear to have a weak effect on the association among gp120 subunits in the trimeric context because the V1 and V2 loops are oriented below the gp120 outer domain while protruding away from the central threefold axis of the Env trimer. However, the relatively short L1 loop (residues 162–168) has been shown to make a substantial contribution to associations among the gp120 subunits in the Env trimer.^6,13,14^ The observed significantly higher flexibility of this short loop in R2-gp120 may make it more easy to disrupt the inter-subunit interactions, thus increasing the probability of opening the trimer crown even when unliganded. Both V4 and V5 loops are longer and exhibit significantly higher flexibility in R2-gp120 than in H061.14 gp120. Because V4 and V5 are connected to β20 (one of bridging-sheet elements) and α5 (layer 3) through rigid β19 and β24, respectively, it is very likely that dynamical behaviours of these two surface loops may mediate/modulate conformational dynamics of the β20-β21 hairpin and layer 3, respectively. The stronger mobility of V4 and V5 in R2-gp120 likely makes a larger contribution to increasing fluctuations of β20-β21 and α5, respectively, which have been shown important for triggering the formation of bridging sheet^16,58,59^ and for communication between inner and outer domains,^15,16^ respectively.

Based on comparative HDX analyses of Env trimers free in solution and in complex with CD4 binding-site-targeted inhibitors, Guttman *et al.*^16^ proposed two distinct allosteric networks engaged in CD4-induced conformational changes of Env: the “opening” network, which involves breakage of inter-protomer interactions at the trimer apex and repositioning of the bridging-sheet elements and V1/V2 region, can open the Env crown and expose the coreceptor binding site; the “priming” network, which connects the CD4 binding site to the HR1 of gp41 *via* layers 1 to 3 of the gp120 inner domain, is responsible for inter-subunit communication and priming the gp41 fusogenic properties. Of note is that most of the structural regions involved in these two networks were observed to have higher flexibility in R2-gp120 than in H061.14-gp120, implying more active networks of R2-Env. It could be anticipated that the highly active opening and priming networks in R2-Env may make it easier to open the trimer crown, expose the coreceptor binding site, and trigger the metastable gp41-entry machinery even in the absence of CD4.

### Collective motions

To extract the largest-amplitude collective motions during MD simulations, PCA was performed on the joined equilibrium trajectories. The values of the total mean square fluctuations (TMSF) of H061.14-gp120 and R2-gp120 are 33.5 and 48.5 nm^2^, respectively, indicating that the latter experienced significantly larger atomic fluctuations than the former during simulations. This is in agreement with the above comparison of average RMSF values, both revealing the enhanced mobility/flexibility of R2-gp120 when compared to H061.14-gp120.

**Fig. 5:**
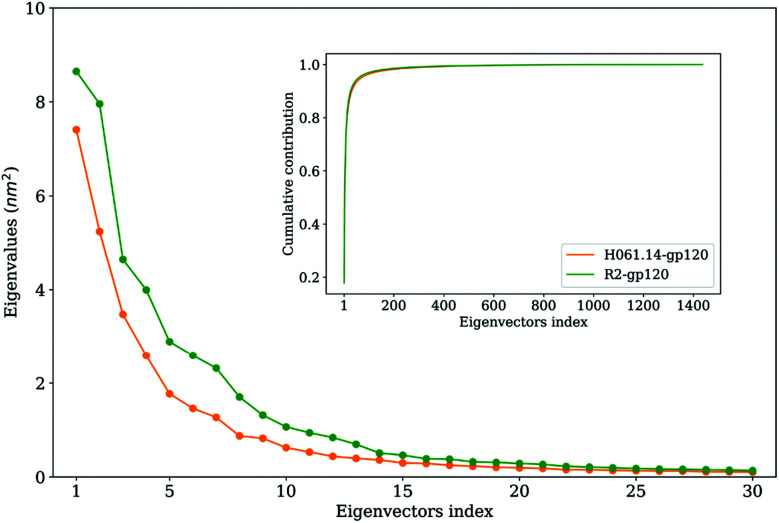
Eigenvalues of the first 30 eigenvectors (main plot) and cumulative contribution of all eigenvectors to the total mean square fluctuations (inset plot) for the H061.14-gp120 (orange line) and R2-gp120 (green line).

Fig. 5 shows the eigenvalues as a function of eigenvector index and the cumulative contribution of eigenvectors to TMSF. It is clear that for both gp120s, their eigenvalues decrease rapidly until the eigenvector index increases to 10. Nevertheless, for R2-gp120, its first 10 eigenvectors have significantly larger eigenvalues than the corresponding eigenvectors of H061.14, implying that the former experienced larger-amplitude fluctuations than the latter along these eigenvectors. Moreover, for H061.14-gp120 and R2-gp120, the cumulative contributions of the first three and 10 eigenvectors to TMSF are 43.8% and 48.1%, and 76.1% and 77.5%, respectively, indicating that the first 10 eigenvectors, especially the first three eigenvectors, span an essential subspace within which the largest collective motions take place. We note that R2-gp120 requires a fewer number of eigenvectors to reach the same level of cumulative contribution than H016.14-gp120, which is a common feature of a flexible protein (or the form of a protein with higher flexibility) when compared to its rigid homologue (or the form of the protein with higher rigidity).^57,60–62^

**Fig. 6:**
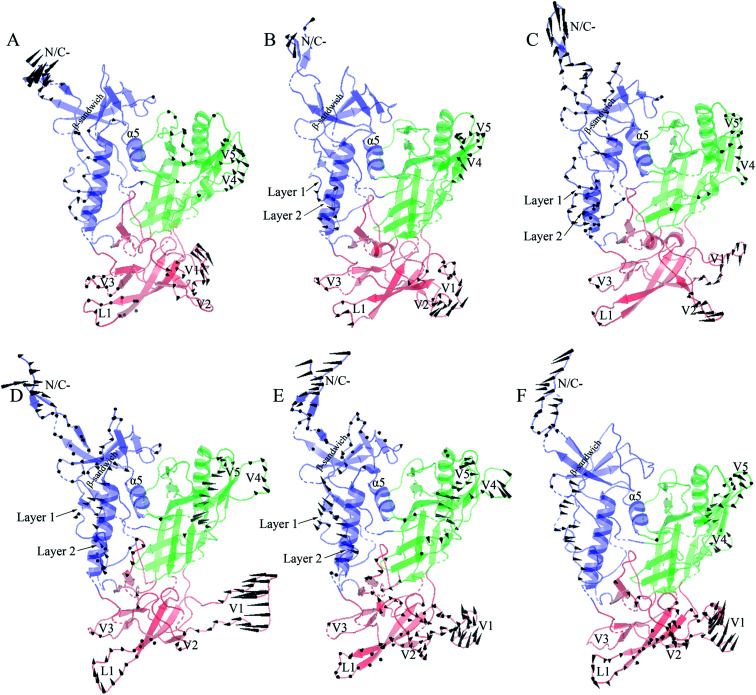
Porcupine plots showing the largest-amplitude collective motions of the two gp120 structural models. (A)–(C) Modes of collective motions of H061.14-gp120 along the eigenvectors 1–3, respectively. (D)–(F) Modes of collective motions of R2-gp120 along the eigenvectors 1–3, respectively. In these plots, the direction and length of the cone drawn on a C_α_ atom represent the fluctuating direction and amplitude of this atom, respectively, along the corresponding eigenvector; the inner domain, outer domain, and small domain are coloured blue, green, and red, respectively.

Fig. 6 shows the collective motions of H061.14-gp120 and R-gp120 along the first three eigenvectors in terms of porcupine plots in which the cone was drawn on the C_α_ atom, with its pointing direction and length representing the direction and amplitude of the C_α_ fluctuation, respectively. As shown in Fig. 6, the largest-amplitude collective fluctuations of both gp120s mainly involve the N-, C-termini, some substructures in the inner domain, the outer-domain surface loops (V4 and V5), and some elements in the small domain, whereas the structural core, including α5 from the inner domain and β-barrels from the outer domain, are rarely involved in collective motions. Nevertheless, close inspection reveals that the inner and small domains of R2-gp120 span more substructures involved in collective motions with larger displacement amplitudes than those of H061.14-gp120, possibly implying a greater potential for R2-gp120 to transition out of the unliganded state in the absence of CD4 (discussed below).

In the case of the neutralization-sensitive, CD4-independent R2-gp120, the collective outward shifts of layers 1 and 2 along the first (Fig. 6D) and second eigenvectors (Fig. 6E) will enlarge the cavity located between the inner and outer domains, allowing β20-β21 hairpin to move in the same direction as layers 1 and 2. Previous studies^37,40,58^ have suggested that the opening of inter-domain cavity followed by the rearrangement of β20-β21 hairpin is a crucial initial step for triggering gp120's conformational transition from the unliganded to the CD4-bound state since the reorientation of β20-β21 results in the formation of the mature CD4-Phe43-binding pocket and the disruption of hydrogen bonds between β3 and β21, which is a prerequisite for further repositioning of V1/V2 and the formation of the mature bridge sheet. In the motional mode along the eigenvector 3 (Fig. 6F), β21 and the four β-stands (βA to βD) within the V1/V2 region move collectively outwards and downwards, thus likely allowing for a full extension and reorientation of these substructures which, although not observed in our simulations due to limited simulation time, were considered as crucial events in gp120 conformational transition.^58^ Of interest is that the homology model showing a full-extension downward orientation of V1/V2 relative to gp120 core has been constructed by Langley *et al.*,^37^ who suggest that the CD4-independent gp120 has a higher probability to adopt this intermediate state than CD4-dependent gp120 when unliganded by CD4. In addition, the bending and twisting motions of the V1/V2 region along the eigenvectors 1 and 2, respectively, may help to disrupt its adhesion with the V3 loop, which is fully exposed in the CD4-bound state of gp120 ^17,63^ and, upon exposure, participates in the formation of the mature coreceptor-binding site or of the CD4i epitope.^64,65^ Also worth noting is the collective fluctuations of the β-sandwich in the inner domain of R2-gp120 (Fig. 6D and E). Because the β-sandwich has been considered as a hub from which the layers 1 to 3 emanate,^15^ its mixed twisting and bending motions (along eigenvectors 1 and 2) may facilitate the regulation of relative orientations of the three layers with respect to one another, thus being beneficial to conformational changes of gp120.

In the case of the neutralization-resistant, CD4-dependent H061.14 gp120, the collective outward shifts of the lower halves of layers 1 and 2 were observed along the second eigenvector (Fig. 6B). Although such shifts will enlarge to a certain extent the inter-domain cavity, no apparent movement was observed on the β20-β21 hairpin. For all the three motional modes displayed, the collective shifts in V1/V2 region mainly involve loops L1, V1, and V2 rather than βA to βD. Although collective fluctuations of these three loops could perturb the association of V1/V2 with V3 loop, they may not cause the full extension and repositioning of the entire V1/V2 region. Only along the third eigenvector can collective fluctuations of the layers 1 and 2 and the β-sandwich be observed (Fig. 6C). However, the common shifts of the lower halves of layers 1 and 2 towards the outer domain will narrow the inter-domain cavity, thus likely preventing the rearrangement of β20-β21 and favouring the maintenance of the unliganded state of gp120.

To this end, it can be concluded that differences in the moving direction, fluctuating amplitude, and spanning range of the substructures involved in collective motions between R2-gp120 and H061.14-gp120 likely lead to different consequences with respect to conformations of gp120s, *i.e.*, a higher capacity for H061.14-gp120 to maintain the unliganded state and a greater potential for R2-gp120 to transition to the CD4-bound state, in which the fully exposed V3 loop and the mature bridging sheet allow for high-affinity interaction with the coreceptor and for efficient recognition by various CD4i or CD4bs neutralizing monoclonal antibodies.^43^ The observed collective motional modes of R2-gp120 are consistent with the hypothesis that the phenotype of the CD4-independent infectivity/high neutralization sensitivity is related to the increased capacity of spontaneous exposure of coreceptor-binding site and specific neutralizing epitopes.^12,43^ In fact, the cryo-electron tomography of the trimeric Env from a CD4-independent SIV strain shows a constitutively “open” state in which the three gp120 protomers splay out in a conformation similar to the CD4-bound state,^66^ which is susceptible to antibody neutralization and capable of interacting with coreceptor.

### Free energy landscapes

**Fig. 7:**
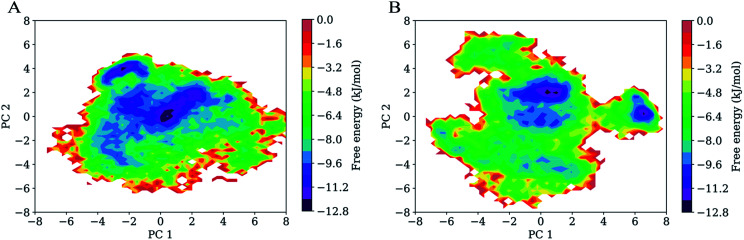
Free energy landscapes (FELs) of the two gp120 structural models as a function of the projection of the joined equilibrium trajectory onto the first (PC1) and second (PC2) principal components. (A) Two-dimensional FEL of H061.14-gp120. (B) Two-dimensional FEL of R2-gp120. The colour bar represents the relative free-energy level in unit of kJ mol^−1^.

Fig. 7 shows FELs as a function of the projection of the joined equilibrium trajectory onto the essential subspace spanned by PC1 and PC2. Overall, the FEL of H061.14-gp120 is regular and continuous, exhibiting an oval-like shape (Fig. 7A), whereas the FEL of R2-gp120 is irregular and divergent (Fig. 7B), presenting a shape more complicated than that of H061.14-gp120's FEL; in addition, the FEL of R2-gp120 covers a larger region in the essential subspace than that of H061.14-gp120. The above differences imply that R2-gp120 has larger conformational entropy and more complex kinetic behaviour than H061.14 gp120.

For both gp120 models, there are two basins that have the lowest free-energy level (≤−10.4 kJ mol^−1^), indicating two main conformational substates sampled during MD simulations. However, the two main substates of H061.14-gp120 are separated by lower barriers and could be connected by shorter transition pathways than the two main substates of R2-gp120, implying that these two substates of H061.14-gp120 can interconvert more easily and are more similar to each other than those of R2-gp120. In fact, in the FEL of H061.14 gp120, most of the local minima with free-energy level lower than −6.4 kJ mol^−1^ are localized within a single large global free energy minimum basin, whereas the free-energy surface of R2-gp120 is more rugged and contains three distinct basins with free-energy level lower than −6.4 kJ mol^−1^. This indicates that R2-gp120 sampled more conformational substates that are characterized by similar free-energy levels but large conformational differences; therefore, it seems that the neutralization-sensitive, CD4-independent R2-gp120 is more inclined to escape the unliganded basin than the neutralization-resistant, CD4-dependent H061.14-gp120. Furthermore, it appears that most of the local minima within the single large basin of H061.14-gp120 are characterized by relatively lower free-energy levels than those within the three basins of R2-gp120, implying a lower thermostability of R2-gp120. This on the one hand explains the higher conformational flexibility of R2-gp120, on the other hand enhances its probability of transition from the unliganded to the CD4-bound state.

The characteristic differences in FELs between R2-and H061.14 gp120 are in line with the differences between FEL models proposed by D'Amico *et al.* for explaining the flexibility–stability–activity relationships in extremophilic enzymes.^67^ Specifically, when compared to the funnel-like FEL of a stable and rigid protein (*e.g.*, H061.14-gp120 or thermophilic enzyme), the FEL of its flexible, unstable counterpart (*e.g.*, R2-gp120 or psychrophilic enzyme) is characterized by a shallower depth, wider width, and more rugged bottom which comprises more local minima (*i.e.*, conformational states/substates) with higher free-energy levels and lower inter-minima barriers. It has been argued that the more the local minima at the bottom of FEL, the higher the probability that the protein can sample the ligand-association competent states/substates;^67,68^ upon ligand association, the low barriers between minima also make it easy to shift conformational equilibrium toward the ligand-bound state (*i.e.*, to deepen the free energy well within which the ligand-bound state resides).^69,70^ Therefore, a highly flexible protein as compared to its rigid homologues is more advantageous in interacting with multiple structurally dissimilar ligands and in modulating both the thermodynamics and kinetics of protein-ligand recognition/binding, thus being easier to be activated by ligands.^61,71^

It should be pointed out that the FELs constructed based on the combined method of PCA and probability density function are incomplete and feature a low-resolution character due to the limited conformational sampling and large dimensionality reduction. However, the detailed comparison still reveals differences in the thermodynamics (distributions of the sampled conformational states/substates) and kinetics (conversion between states/substates) between R2- and H061.14-gp120. Taken together, it can be concluded that R2-gp120 has larger conformational entropy, richer conformational diversity, and lower thermostability than H061.14 gp120, all of which act together to improve its capability to spontaneously sample the CD4-bound or near-CD4-bound states. Both smFRET^11^ and HDX^12^ data support the viewpoint that CD4i antibodies (such as 17b) bind gp120 *via* conformational selection:^72^ the different conformations (*i.e.*, unliganded, CD4-bound, and various intermediate states) of the ligand-free gp120 coexist in equilibrium with distinct population distributions, and 17b can bind selectively to the CD4-bound or near-CD4-bound states, shifting the equilibrium toward the bound state. For H061.14-gp120, we consider that the poor conformational diversity and high thermostability limits the spontaneous sampling of the CD4-bound conformation, but binding of CD4 to its unliganded state can trigger/induce transition to the CD4-bound state. This speculation is supported by the experimental work by Guttman *et al.*,^12^ who showed that the binding of 17b to KNH1144 SOSIP.664 trimers occurs very slowly in the absence of CD4 but rapidly in the presence of CD4. In the case of R2-gp120, its high conformational flexibility and low thermostability increase the probability of spontaneously sampling the conformations which are suitable for selective bindings of antibodies/coreceptor, and by doing so they stabilize the CD4-bound state. Of note is that although coreceptor/CD4i antibodies bind only to the CD4-bound conformation of gp120, H061.14-gp120 may be difficult to reach this state unless induced by CD4, while R2-gp120 can spontaneous sample multiple conformational states/substates, thus increasing the opportunity for the selective binding by antibodies/coreceptor.

## Conclusions

In this paper, we constructed two unliganded gp120 structural models from HIV-1 isolates differing in the phenotype of CD4-dependency/neutralization sensitivity and further performed multiple-replica MD simulations to investigate the differences in dynamics and energetics between these two models. Comparative analyses of the joined equilibrium MD trajectories reveal that the CD4-independent, neutralization-sensitive R2-gp120 is more structurally unstable and conformationally flexible than the CD-dependent, neutralization-resistant H061.14-gp120. In particular, the structural regions involved in the proposed opening and priming networks were observed to have significantly higher flexibility in R2-gp120 than in H061.14-gp120, and this likely exerts larger perturbations on associations between gp120 subunits and between gp120 and gp41 in R2-Env trimer, thus making it easier to open the trimer crown and prime the fusogenic properties of gp41. Comparison between molecular motions of these two gp120s indicates that there are more substructures that exhibit larger amplitudes of collective displacements in R2-gp120 than in H061.14-gp120. Of interest is that the differences in moving directions of some substructures (such as layers 1 and 2 in the inner domain and structural elements in the small domain) will lead to different conformational consequences: a higher capacity for H061.14-gp120 to maintain the neutralization-resistant unliganded state while a greater potential for R2-gp120 to transition to the neutralization-sensitive CD4-bound state. The constructed FEL of R2-gp120 exhibits a larger, more rugged and complicated free-energy surface, and a generally higher free-energy level of most local minima than that of H061.14-gp120, indicating that R2-gp120 has larger conformational entropy, richer conformational diversity and more complicated kinetic behaviour, and lower thermostability than H061.14-gp120. Collectively, it can be concluded that the unliganded form of R2-gp120 is more inclined to transition to the CD4-bound state in the absence of the induction by CD4 than that of H061.14-gp120.

In the HIV-1 Env trimer, gp120 is not only the crucial subunit responsible for interactions with the receptor and coreceptor, but also the important target for recognition by most of the Env-directed antibodies. When compared to the unliganded form of H061.14-gp120, the lower structural stability and higher conformational flexibility of the unliganded R2-gp120, in conjunction with its higher propensity to transition to the CD4-bound state, could impart R2-Env with increased capacity to sample the open state in the absence of CD4, in which the coreceptor-binding site and the conserved antibody neutralization epitopes have been formed and exposed, thus allowing for efficient recognition/binding by relevant antibodies and coreceptor *via* conformational selection. On the contrary, H061.14-gp120 exhibits the lower conformational flexibility and higher propensity to maintain the unliganded state, and this makes H061.14-Env less able to reach the active open state unless induced by CD4. Our comparative MD simulations and FEL construction reveal the differences in dynamics and energetics between R2- and H061.14-gp120, and explain why HIV-1 isolates R2 and H061.14 have differential phenotypes of CD4-dependency/neutralization sensitivity. Because of the characteristics of instability and spontaneous opening propensity, the neutralization-sensitive, CD4-independent Env trimer appears not to be a good candidate for rational design of HIV-1 immunogen. Modifications to the neutralization-resistant, CD4-dependent Env trimer for reducing CD4-induced opening are likely to be a good strategy because the ultra-stable closed Env mimic can be sufficiently long-lived to elicit antibodies that recognize the dominant closed state of Envs on infectious virus.

## Conflicts of interest

There are no conflicts to declare.

## Supplementary Material

RA-008-C8RA00425K-s001
