# Supplementary material for: Insights into the molecular mechanism underlying CD4-dependency and neutralization sensitivity of HIV-1: a comparative molecular dynamics study on gp120s from isolates with different phenotypes
Source: RSC Adv. 2018 Apr 17;8(26):14355–68. doi: 10.1039/c8ra00425k (PMC9079880; doi:10.1039/c8ra00425k)
Supplement: RA-008-C8RA00425K-s001 [file RA-008-C8RA00425K-s001.pdf]

Electronic Supplementary Material (ESI) for RSC Advances.
This journal is © The Royal Society of Chemistry 2018

Supplementary information

Insights into molecular mechanism underlying CD4 dependency and neutralization sensitivity of HIV-1: A comparative molecular dynamics study on gp120s from isolates with different phenotypes

Yi Li,[†a] Lei Deng,[†a] Shi-Meng Ai,[b] Peng Sang,[c] Jing Yang,[a] Yuan-Lin Xia,[a] Zhi-Bi Zhang,[a] Yun-Xin Fu,[a,d*] Shu-Qun Liu[a*]

a. State Key Laboratory for Conservation and Utilization of Bio-Resources in Yunnan, Yunnan University, Kunming, P.R. China

b. Department of Applied Mathematics, Yunnan Agricultural University, Kunming, P. R. China

c. College of Agriculture and Biological Science, Dali University, Dali, P. R. China

d. Human Genetics Center and Division of Biostatistics, School of Public Health, the University of Texas Health Science Center, Houston, USA

† These authors contributed equally to this work.

* Correspondence: Yunxin.Fu@uth.tmc.edu (YXF) shuqunliu@ynu.edu.cn (SQL)

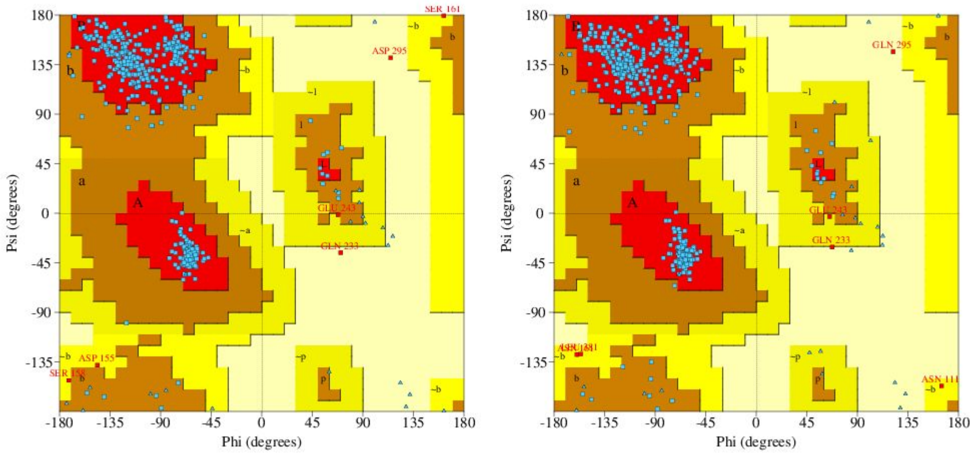

Ramachandran Plot statistics

	No. of residues	%-tage
Most favoured regions [A,B,L]	380	91.3%
Additional allowed regions [a,b,l,p]	30	7.2%
Generously allowed regions [~a,~b,~l,~p]	4	1.0%
Disallowed regions [XX]	2	5%
Non-glycine and non-proline residues	416	100%
End-residues (excl. Gly and Pro)	2	
Glycine residues	26	
Proline residues	23	
Total number of residues	467	

Ramachandran Plot statistics

	No. of residues	%-tage
Most favoured regions [A,B,L]	385	91.0%
Additional allowed regions [a,b,l,p]	32	7.6%
Generously allowed regions [~a,~b,~l,~p]	4	0.9%
Disallowed regions [XX]	2	0.5%
Non-glycine and non-proline residues	423	100.0%
End-residues (excl. Gly and Pro)	2	
Glycine residues	31	
Proline residues	23	
Total number of residues	479	

Fig. S1 Ramachandran plots of the constructed structural models of H061.14- (left) and R2-gp120 (right).

Table S1. Validation results of the constructed gp120 structural models (H061.14- and R2-gp120) and template (PDB ID: 5FYJ, chain G).

Structure or model	PROVE[a]	VERIFY3D[b]
5FYJ, chain G	74 (5%)	85.26%
H061.14-gp120	93 (6.1%)	79.66%
R2-gp120	101 (6.5%)	84.76%

[a]The total number of buried outlier protein atoms (percentage is in parentheses).

[b]Percent of the residues had an averaged 3D-1D score >= 0.2.

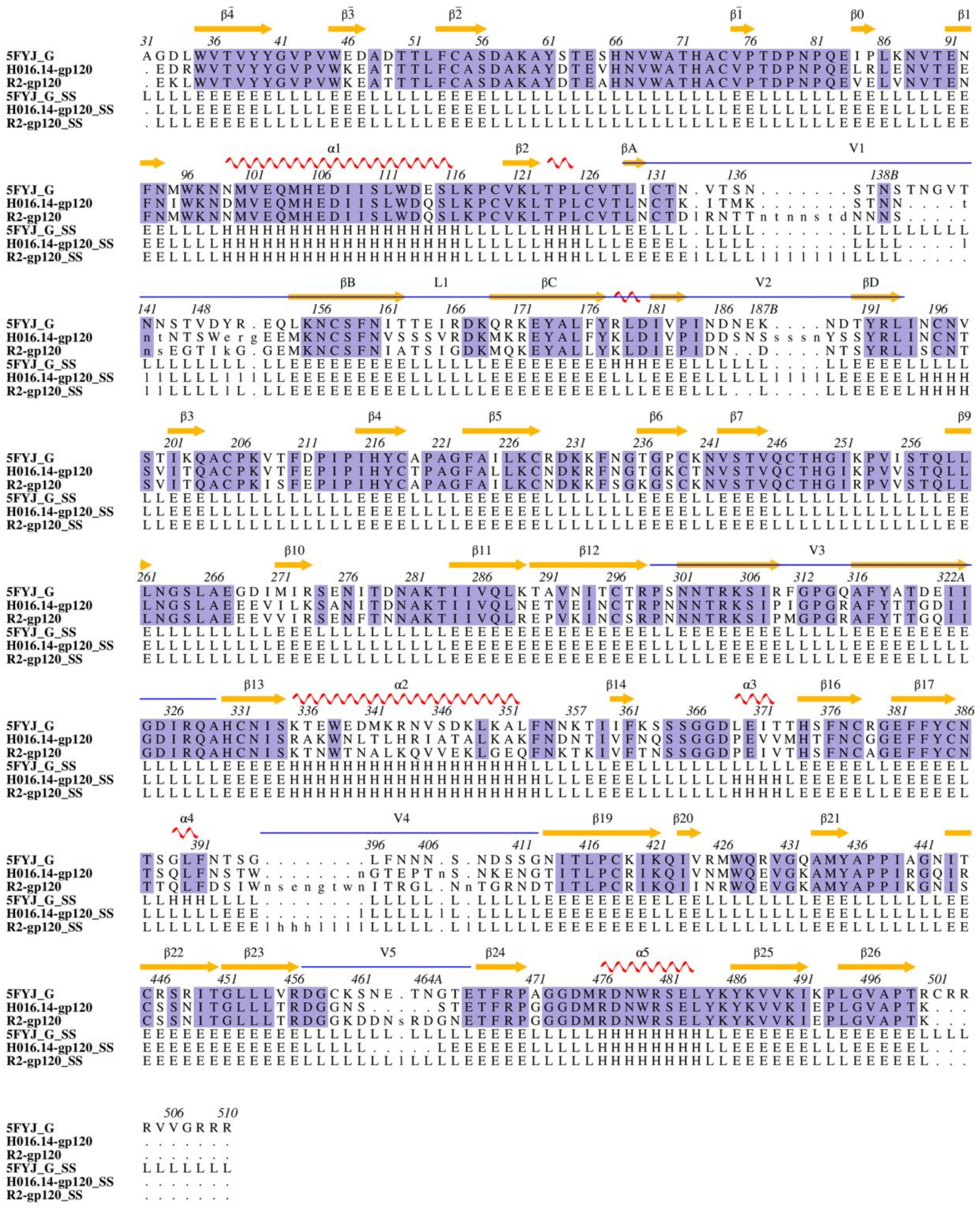

Fig. S2. Structure-based multiple sequence alignment among the template (PDB ID: 5FYJ, chain G, labelled as 5FYJ_G), H061.14-, and R2-gp120. Conserved residues are shaded in light blue. Secondary structure (SS) is shown below the sequence alignment, with H/h, E/e, and L/l representing the α-helix (or 3/10 helix), β-strand, and loop, respectively. Regular secondary structural elements of the template are numbered according to the HXBc2 crystal structures (PDB IDs: 3JWD and 1G9M), with orange arrows and red spiral representing β-strands and α-helices (or 3/10 helices), respectively. The variable regions of V1/V2, V3, V4, and V5 are denoted above the alignment by blue line segments. The four β-strands in the V1/V2 region are labelled βA to βD, respectively, and the loops located between βA and βB, between βC and βD, and between βB and βC are labelled V1, V2, and L1, respectively.
